# The Value of Adenosine Deaminase 2 in the Detection of Tuberculous Pleural Effusion: A Meta-Analysis and Systematic Review

**DOI:** 10.1155/2022/7078652

**Published:** 2022-09-10

**Authors:** Tingting Zeng, Bing Ling, Xueru Hu, Shuyan Wang, Wenliang Qiao, Lijuan Gao, Yongchun Shen, Dajiang Li

**Affiliations:** ^1^Department of Respiratory and Critical Care Medicine, West China Hospital of Sichuan University and Division of Pulmonary Diseases, State Key Laboratory of Biotherapy of China, Chengdu 610041, China; ^2^Lung Cancer Center, West China Hospital of Sichuan University, Chengdu 610041, China; ^3^Center of Infectious Diseases, West China Hospital of Sichuan University, Chengdu 610041, China

## Abstract

Adenosine deaminase 2 (ADA_2_) is reported as a novel diagnostic biomarker for tuberculous pleural effusion (TPE) in many studies. This meta-analysis was conducted to systematically evaluate the general diagnostic performance of pleural ADA_2_ in TPE. After searching for relevant studies that investigated the diagnostic performance of pleural ADA_2_ in TPE in several databases, we assessed and selected eligible studies to calculate pooled parameters by STATA 16.0 software. A final set of thirteen studies entirely met the inclusion standards and were used to calculate pooled parameters in our meta-analysis. Among them, there were nine English studies and four Chinese studies. The pooled parameters of pleural ADA_2_ in diagnosing TPE were summarized as follows: sensitivity, 0.91 (95% CI: 0.86–0.95); specificity, 0.93 (95% CI: 0.92–0.95); positive likelihood ratio, 13.9 (95% CI: 10.6–18.3); negative likelihood ratio, 0.09 (95% CI:0.06–0.16); diagnostic odds ratio, 147 (95% CI: 76–284); and the area under the curve, 0.95 (95% CI: 0.93–0.97). Pleural ADA_2_ is a reliable indicator with excellent accuracy in TPE diagnosis. However, we need to combine pleural ADA_2_ with diverse examinations to diagnose TPE in clinical practice.

## 1. Introduction

Tuberculosis (TB) is the most common cause of morbidity and mortality in many low-income and middle-income countries, with an estimated 10.0 million cases in 2019, according to the World Health Organization (WHO) [1]. In adult patients infected with *Mycobacterium tuberculosis*, extrapulmonary tuberculosis accounts for 25% of the disease. The pleura is the second leading site of extrapulmonary tuberculosis, next to the lymph nodes [2]. The involvement of the pleura by *Mycobacterium tuberculosis* can result in the generation of excessive pleural effusion, also named as tuberculous pleural effusion (TPE).

Pleural effusion is common in clinical contexts and can be induced by diverse primary diseases, such as malignant tumors, pneumonia, tuberculosis, congestive heart failure, and pulmonary embolism [3]. Although TPE is common in the clinical context, its diagnostic confirmation is still intricate. The gold standard for TPE diagnosis is microbiology or biopsy [4]. Nevertheless, these conventional methods are not always helpful in identifying TPE since they have some limitations. Culturing *M. tuberculosis* can offer 100% diagnostic specificity. However, culturing pleural effusion has a relatively low positive rate (approximately 25–37%), and it usually takes several weeks [5]. Pleural biopsy is an invasive procedure with various complications that require extended expertise and precision equipment. However, it cannot be carried out in all hospitals [4, 6]. Therefore, it is vital to explore and develop less invasive diagnostic methods.

Currently, pleural adenosine deaminase (ADA) is widely applied to examine tuberculous pleural effusion (TPE) [7]. ADA is distributed among various human tissues, and it can convert adenosine to inosine [8, 9]. This enzyme consists of three forms: ADA_1_, ADA_1+cp_, and ADA_2_. Studies have shown that pleural ADA and its isozymes apparently increase in TPE, ADA_2_ is more specific than total ADA, and ADA_2_ occupies the majority of the total ADA activity [8]. Mohammadtaheri et al. reported that the diagnostic accuracy of pleural ADA and ADA_2_ in TPE was 88% and 93.5%, respectively. The isoenzyme ADA_1_ increased in both monocytes and lymphocytes, whereas ADA_2_ was derived from monocytes-macrophages [10]. Patients with parapneumonia, lymphatic gland tumors, malignant tumors, and collagen vascular disease (related to the cellular activation of lymphocytes) have a high level of pleural ADA, which causes frequent false-positive results [5]. So, separated ADA isozymes may help distinguish the root of pleural effusion. Most studies have suggested that pleural ADA is a reliable biomarker for differentiating TPE from other effusions [11], but there is little information about ADA_2_ alone. Therefore, to illustrate the potential of pleural ADA_2_ to detect TPE, we comprehensively reviewed and analyzed the available literature.

## 2. Methods

### 2.1. Literature Search Strategy

We performed this meta-analysis under the guidance of the Preferred Reporting Items for Systematic Reviews and Meta-Analyses (PRISMA) [12]. Embase, Web of Knowledge, PubMed, CNKI, WEIPU, and WanFang databases were searched by two investigators (TZ and BL) independently for primary articles that investigated the diagnostic value of ADA_2_ in TPE and that were published until November 2021. The search strategy was made as follows: “adenosine deaminase or ADA or adenosine deaminase isoenzymes or ADA isoenzymes or ADA_2_” and “tuberculosis” and “pleural effusion or pleural fluid or tuberculous pleurisy” and “sensitivity or specificity or accuracy.” We even manually searched the references or relevant meta-analyses to identify other potential studies.

### 2.2. Study Selection

The same two reviewers (TZ and BL) assessed all the literature independently to find qualified studies. Once there was any divergence, the agreement was reached by negotiation. Our meta-analysis contained a final set of studies that fit the following criteria: (1) literature type: an original study that reported diagnostic specificity and sensitivity to form the complete diagnostic two-by-two table and written in Chinese or English; (2) diagnostic methods: a study that used the gold standard for definitive diagnosis and estimated the diagnostic ability of ADA_2_ in TPE. Excluding studies were as follows: conference abstracts, editor's comments, duplicate studies, and studies containing fewer than 20 patients.

### 2.3. Study Quality Assessment and Data Abstraction

Two authors (TZ and BL) applied the Quality Assessment of Diagnostic Accuracy Studies-2 tool (QUADAS-2) to evaluate the methodological quality of the studies [13, 14], which involved four domains for assessments. The RevMan 5.3 software was used to make the quality evaluation form.

After browsing all the selected studies, TZ and BL extracted several critical data using a standardized extraction form. The form is composed of several elements as follows: authors, publication year, country, testing method, gold standard, cutoff value, and 2 × 2 contingency tables. A consensus-based discussion was held if any disagreements occurred in this assessment procedure.

### 2.4. Statistical Analysis

We extracted critical data from these eligible studies and then calculated TP, TN, FP, and FN. All data were collected and summarized in Excel for future analysis. Review Manager 5.4 (The Cochrane Collaboration, Copenhagen, Denmark) was used to plot the study selection flowchart. STATA 16.0 (Stata Corp., College Station, TX) was recommended for deep statistical testing. A bivariate random-effect model was performed to calculate merged estimates of sensitivity, specificity, positive likelihood ratio (PLR), negative likelihood ratio (NLR), and diagnostic odds ratio (DOR), and their 95% confidence intervals (CIs) [15]. A summary receiver operating characteristic (SROC) curve was constructed to determine whether a threshold effect existed in the study. The area under the curve (AUC) was also calculated to demonstrate the integrated diagnostic value of ADA_2_ [6, 16]. There were inevitable variations among studies, and these variations may lead to significant heterogeneity. *P* < 0.05 or the inconsistency index (I^2^) ≥50% illustrated that heterogeneity existed among studies apparently. The meta-regression analysis was essential to seek a possible source of heterogeneity. The following covariates were considered as possible sources of heterogeneity: publication year (before 2010 vs. after 2010), country (China vs. others), subject (<100 vs. ≥100), testing method (Giusti's method vs. others), and ADA_2_ cutoff value (<30 U/L vs. ≥30 U/L). Deeks' funnel plot was made by STATA 16.0 software to detect publication bias in the included studies [17]. To better interpret the clinical sense of measuring ADA_2_, the Fagan nomogram was drawn to compute posttest probability (PTP). A two-sided test was used in the statistical analysis, and *P* < 0.05 was considered significant.

## 3. Results

**Figure 1:**
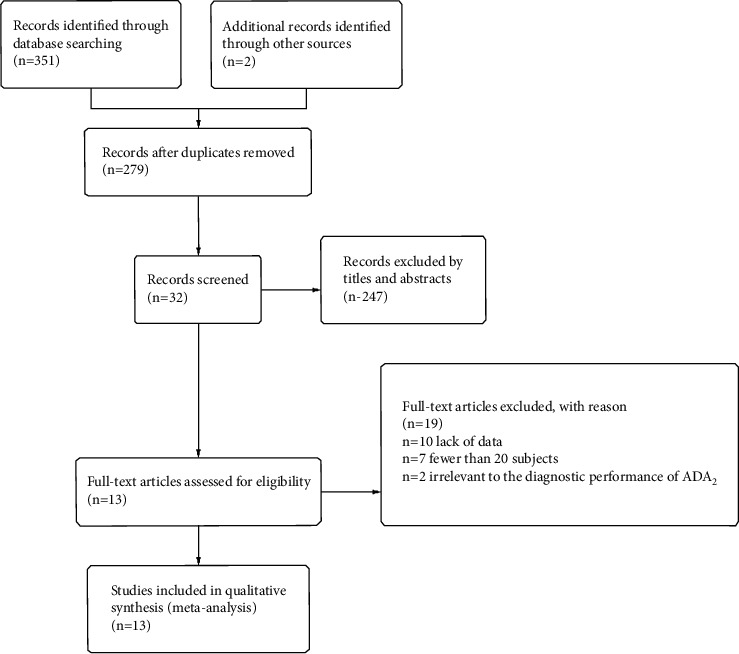
PRISMA flowchart of the selection process.

According to the search strategy, the preliminary search generated 353 articles. Seventy-four duplicate articles were removed after comparison. By screening the titles and abstracts, 247 articles were removed because they were not original studies or irrelevant to our analysis. We excluded 19 articles after carefully reading the details of the articles; the reasons for deletion were that they had no concern with the diagnostic performance of ADA_2_, they involved fewer than 20 subjects, or they had too little data to make sense. Only 13 eligible studies met the inclusion criteria [18–30]. Among them, there were nine English studies and four Chinese studies. The study selection process is shown in Figure 1.

### 3.1. Study Characteristics

Table 1 generalizes the details of the 13 studies, including 921 TPE patients and 1,409 non-TPE controls (an average of 180 subjects for each study, ranging from 34 to 879). Two studies only made clinical diagnosis, evidenced by clinical symptoms, radiology, pleural effusion detection, and the reaction to antituberculous treatment [20, 29]. In comparison, the gold standard method, such as microbiology or biopsy, was performed in the other 11 studies. Seven studies measured ADA_2_ levels using Giusti and Galanti's method; three studies referred to Muraoka's method; one study used enzyme colorimetry; and the remaining two studies did not report such information. Of the 13 studies, one study was retrospective [23], six studies were prospective [21, 24, 26–28, 30], and the others did not report.

### 3.2. Quality of the Included Studies

**Figure 2:**
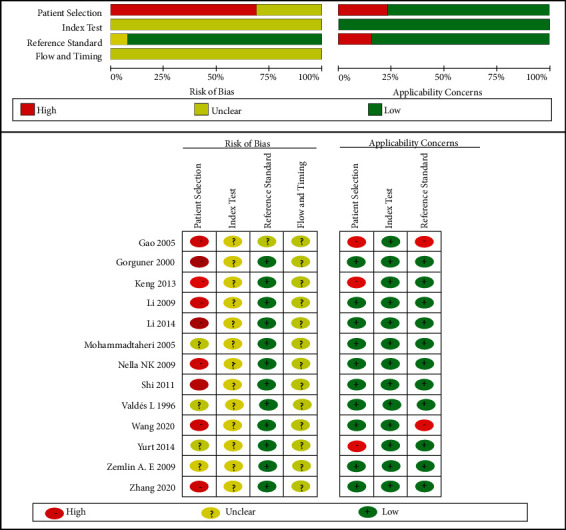
Summary of QUADAS-2 assessments of the included studies. QUADAS-2, Quality Assessment of Diagnostic Accuracy Studies-2. Four fields included are the patient selection methods, index test, reference standard, and flow and timing.

The methodological quality of the 13 eligible studies was assessed by QUADAS-2, as shown in Figure 2. We made different responses to each item on the assessment form. The controversy was resolved by the consensus of the two authors [31]. Most studies had a high risk of bias owing to the selection of patients. For example, ten studies failed to enroll consecutive or random patients [19, 20, 22–27, 29, 30] and did not avoid the case-control study design [18–22, 25, 27, 28]. Almost all studies did not clearly report whether the gold standard test is independent of the ADA_2_ measurement, which may cause diagnostic bias.

### 3.3. Diagnostic Accuracy of Pleural ADA_2_

**Figure 3:**
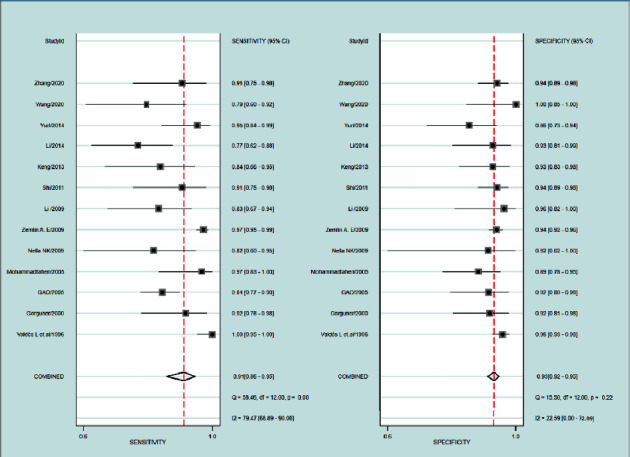
Forest plots of sensitivity and specificity for ADA2. The pooled sensitivity was 0.91 (95% CI: 0.86–0.95), and the pooled specificity was 0.93 (95% CI: 0.92–0.95).

**Figure 4:**
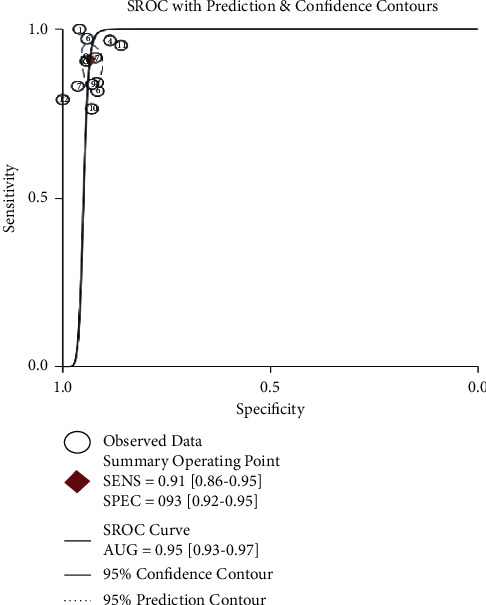
The summary receiver operating characteristic (SROC) curve and the area under the curve (AUC). The AUC was 0.95 (95% CI: 0.93–0.97). AUC, area under the curve; SENS, sensitivity; SPEC, specificity.

**Figure 5:**
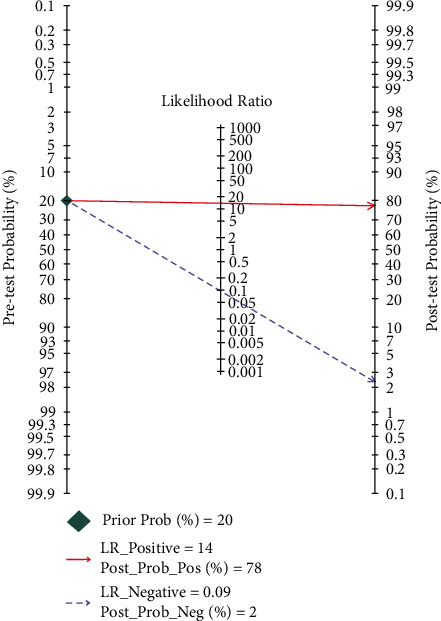
Fagan's nomogram.

After calculating 13 studies, the pooled parameters and their 95% CI were exhibited as follows: the sensitivity was 0.91 (95% CI: 0.86–0.95); and the specificity was 0.93 (95% CI: 0.92–0.0.95). The above two parameters are displayed in the forest plot (Figure 3). The PLR was 13.9 (95% CI:10.6–18.3), and the NLR was 0.09 (95% CI: 0.06–0.16). The DOR was 147 (95% CI: 76–284). The AUC was 0.95 (95% CI: 0.93–0.97) (Figure 4), representing a high level of differential diagnostic ability. Fagan's nomogram for likelihood ratios was used to illustrate the significance of the clinical application of ADA_2_ in TPE (Figure 5); with the estimated prevalence (pretest probability) of TPE in the target population is 20%, if the patients had a positive ADA_2_, the posttest probability of TPE is 78%. While for patients with negative ADA_2_, the posttest probability of them to having TPE is only 2% [32].

### 3.4. Meta-Regression Analysis

**Figure 6:**
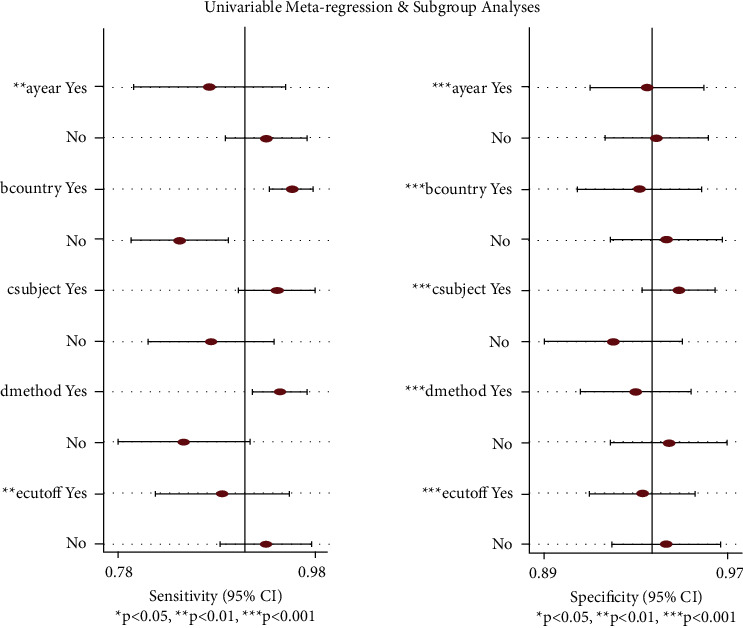
Meta-regression of the included studies. Both sensitivity and specificity were significantly affected by publication year and the cutoff value (*P* < 0.05), indicating that publication year, cutoff value, and other unknown covariates result in heterogeneity.

*P* < 0.05 and *I*^2^ = 87 (95% CI: 74–100) suggested significant heterogeneity existed among included studies, which required further analysis. Therefore, a meta-regression analysis was performed to detect the possible sources of heterogeneity. As listed in Figure 6, specificity was affected by several covariates, such as country, sample size, and testing method, while sensitivity was not. Both sensitivity and specificity were significantly affected by publication year and cutoff value (*P* < 0.05), indicating that the heterogeneity was derived from publication year, cutoff value, or other unknown covariates.

### 3.5. Publication Bias Evaluation

**Figure 7:**
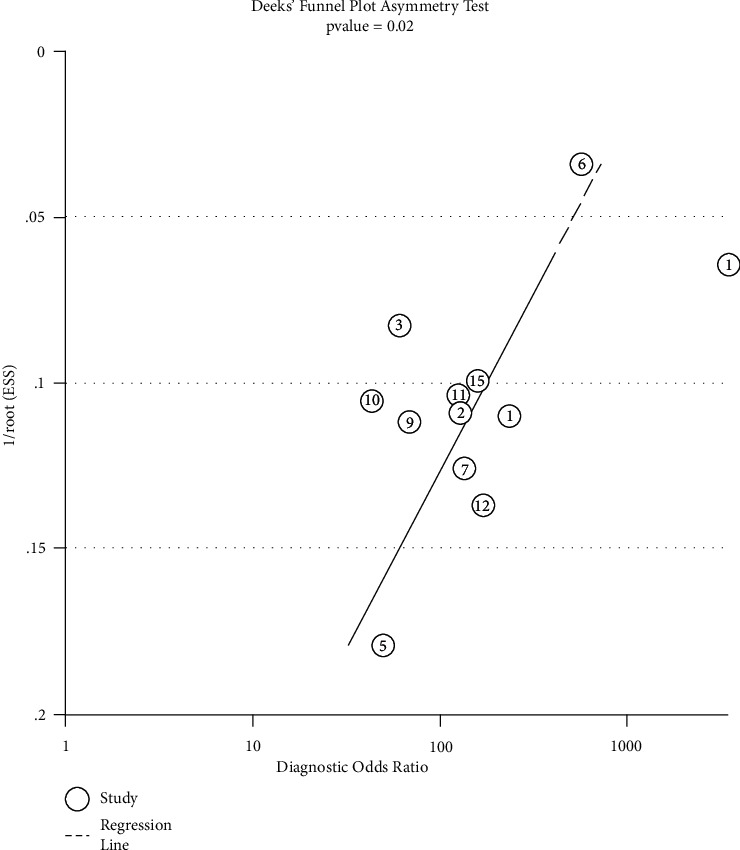
Deeks's funnel plot. The apparent asymmetric shape of Deeks's funnel plot and *P* value = 0.02 indicate that publication bias significantly existed in the study.

The apparent asymmetric shape of Deeks's funnel plot and *P* value = 0.02 indicated that publication bias existed among the included studies (Figure 7).

## 4. Discussion

Considering that conventional diagnostic methods are time-consuming and related to the risk of complications in practice, several biomarkers have been proposed as alternatives to diagnose TPE [20, 26–28]. Among these markers, ADA_2_ receives a lot of attention as a major component in total ADA. Variable diagnostic accuracy has been reported in studies when measuring pleural ADA_2_, prompting us to conduct this meta-analysis to evaluate its actual diagnostic value. Our meta-analysis extracted and pooled the data from 13 eligible studies, and the results suggested that pleural ADA_2_ played a role in differentiating TPE from other types of pleural effusion. However, in the clinic, the diagnosis of TPE should not rely on pleural ADA_2_ alone but in combination with some traditional measurements.

The diagnostic value of ADA in TPE has been extensively investigated, but little attention has been given to pleural ADA_2_ in recent years. Previous studies found that ADA_2_ levels increased in some diseases, such as rheumatoid arthritis, tuberculosis, T cell lymphoblastic malignant tumor, autoimmune liver disease, and acquired immunodeficiency syndrome (AIDS) [33–35]. ADA_2_ exists in monocytes-macrophages and resists immune suppression induced by elevation of adenosine at the time of infection [7, 9, 36]. Several studies assessed the status of pleural ADA_2_ activity in TPE and found that pleural ADA_2_ level accounted for approximately four-fifths of the total ADA level in TPE [37]. Pleural ADA_2_ shows the potential as a diagnostic marker for TPE.

Our results show that pleural ADA_2_ tends to be a sensitive and specific biomarker, with high sensitivity (0.91, 95% CI: 0.86–0.95) and higher specificity (0.93, 95% CI: 0.93–0.95). The sensitivity and specificity are affected by the threshold value. Thus, we plotted the SROC curve to illustrate the overall diagnostic performance [38]. The AUC value was 0.95, indicating excellent test accuracy in our study. DOR is another overall parameter of diagnostic accuracy, reflecting the correlation between diagnosis and disease. The higher the DOR value is, the better the diagnostic efficiency. The mean DOR was 147 (95% CI: 76–284), suggesting excellent discriminate performance. The PLR and NLR were also calculated to make it easier to understand the diagnostic value in the clinical context [39]. PLR > 10 and NLR < 0.1 are strong indicators to confirm and exclude the diagnosis, respectively [40]. The pooled PLR was approximately 14, suggesting that the positive result of the pleural ADA_2_ test in TPE patients was 14-fold higher than in non-TPE patients. In addition, the pooled NLR value of 0.09 indicated that the probability of false-negative of the pleural ADA_2_ test was 9%. Our meta-analysis suggests that pleural ADA_2_ is reliable as an aid in distinguishing TPE from other pleural exudates.

In this meta-analysis, significant heterogeneity (*I*^2^ = 87. 95% CI: 74–100) was found. The meta-regression analysis suggested that the cutoff value and publication year resulted in heterogeneity in both sensitivity and specificity. The pleural ADA_2_ cutoff value ranged from 12 to 60 U/L in these studies. There is no definite standard cutoff value in any country. It changes in different clinical contexts, such as race, testing instruments, laboratory methodologies, disease stages, and history of treatment. Shibagaki et al. reported that standardized antituberculosis management of TPE patients decreased pleural ADA_2_ activity [8]. However, only one study mentioned that the pleural ADA_2_ level was measured after excluding TPE patients who received antituberculosis treatment, which may be one of the reasons for the diversity of the cutoff values [23]. The level of pleural ADA_2_ is also influenced by other factors, such as smoking and MTB load [7]. However, almost all studies did not report the relevant information above. We need to distinguish possible confounding factors when assessing the diagnostic performance of pleural ADA_2_ in TPE in the future. Further studies need to seek an optimal cutoff value in separate clinical contexts and formulate stricter inclusion criteria to avoid bias. However, heterogeneity may be from other sources, such as the study design and blinding method. Our study failed to extract the information above due to the shortage of data.

Our meta-analysis indicates that pleural ADA_2_ is helpful for diagnosing TPE. In addition to ADA_2_, some studies demonstrated that tumor necrosis factor-alpha (TNF-*α*), interleukins (ILs), immunosuppressive acidic protein (IAP), and interferon-*γ* (IFN-*γ*) were also diagnostic biomarkers for TPE [20, 26, 41]. Tianrui-Xue reported that the sensitivity and specificity of the combination of IFN-*γ* and pleural ADA_2_ were 95.03% and 93.47%, respectively [20]. Further investigations are needed to examine the combined diagnostic ability of pleural ADA_2_ with other biomarkers and the value of serum ADA_2_ in TPE patients. Since total ADA measurement is a mature and widely recognized biomarker, pleural ADA_2_ measurement seems not to have technical advantages. However, total ADA can increase in many non-TPE diseases, causing false-positive results. There were studies which reported that pleural ADA_2_ had higher diagnostic accuracy and better specificity than total ADA, especially in Byelorussian patients with tuberculous pleural effusion [21, 42, 43]. So, we should investigate more about pleural ADA_2_ in special patients with TPE in future.

There are several limitations that require attention in our study. First, although we comprehensively searched several databases, only 13 published articles in English or Chinese were included, which may affect the outcome. Second, heterogeneity existed among the studies, apparently. Although we applied meta-regression analysis to seek the possible sources of heterogeneity, the exploration was not enough due to the shortage of data in the included studies. Third, the assessment of methodological quality was incomplete, resulting in an “unclear” QUADAS-2. However, the quality of studies may be one of the sources of heterogeneity. Last but not least, Deeks's funnel plot indicated that publication bias existed among the included studies. The representativeness of the included studies is limited and the positive results are much easier to publish than the negative results, which leads to publication bias and affects the combined effect size of our meta-analysis.

Taken together, pleural ADA_2_ is a reliable indicator with excellent diagnostic accuracy in TPE. Compared with traditional diagnostic methods, it is safe, convenient, and noninvasive. However, we still need to combine pleural ADA_2_ with various examinations to diagnose TPE in current clinical practice.

## 5. Disclosure

The funding agencies were not involved in designing the study, collecting or analyzing the data, writing the manuscript, or making decisions related to the publication.

## Figures and Tables

**Table 1:** A clinical summary of the included studies.

First author	Year	Country	Sample size	Aetiology of non-TPE	Gold standard	Testing method	Cutoff value (U/L)	TP	FP	FN	TN
Valdés [18]	1996	Spain	350	Malignant, parapneumonic, CHF, empyema, and others	Microbiology or biopsy	Giusti's method	40	76	11	0	263
Gorguner [19]	2000	Turkey	87	Malignant and parapneumonic	Microbiology or biopsy	Giusti and Galanti's method	29	33	4	3	47
GAO [20]	2005	China	190	Malignant	Clinical diagnosis	Muraoka's method	37.8	119	4	22	45
Mohammadtaheri [21]	2005	IRAN	93	Malignant, parapneumonic, transudates, and others	Microbiology or biopsy	Giusti and Galanti's method	42	30	7	1	55
Nella [22]	2009	India	34	Malignant	Microbiology or biopsy	Giusti and Galanti's method	60	18	1	4	11
Zemlin [23]	2009	South Africa	879	Malignant, bacterial, empyema, transudates, and others	Microbiology or biopsy + clinical diagnosis	Giusti and Galanti's method	40.6	355	30	10	484
Li [24]	2009	China	64	Malignant	Microbiology + clinical diagnosis	NA	19.7	30	1	6	27
Shi [25]	2011	Taiwan	155	Malignant, parapneumonic, CHF, and others	Microbiology or biopsy + antituberculosis treatment	NA	24	29	7	3	116
Keng [26]	2013	Taiwan	88	Malignant and others	Microbiology or biopsy + clinical diagnosis	Giusti and Galanti's method	12	26	4	5	53
Li [27]	2014	China	90	Malignant	Microbiology or biopsy	Muraoka's method	29.45	36	3	11	40
Yurt [28]	2014	Turkey	93	Malignant and others	Microbiology or biopsy	Giusti's method	20.37	41	7	2	43
Wang [29]	2020	China	52	Malignant	Clinical diagnosis	Enzyme colorimetry	30.4	23	0	6	23
Zhang [30]	2020	China	155	Malignant, parapneumonic, CHF, and others	Microbiology or biopsy + antituberculosis treatment	Muraoka's method	26	29	7	3	116

NA, not available; FN, false-negative; FP, false-positive; TN, true-negative; TP, true-positive; CHF, congestive heart failure.

## Data Availability

The data used to support the findings of the current study are available from the corresponding authors upon request.

## References

[1] Kang W., Yu J., Du J., Yang S., Tang S. (2020). The epidemiology of extrapulmonary tuberculosis in China: a large-scale multi-center observational study. *PLoS One*.

[2] Vorster M. J., Allwood B. W., Diacon A. H., Koegelenberg C. F. N. (2015). Tuberculous pleural effusions: advances and controversies. *Journal of Thoracic Disease*.

[3] Jany B., Welte T. (2019). Pleural effusion in adults-etiology, diagnosis, and treatment. *Deutsches Arzteblatt International*.

[4] Porcel J. M., Azzopardi M., Koegelenberg C. F., Maldonado F., Rahman N. M., Lee Y. C. G. (2015/11/02 2015). The diagnosis of pleural effusions. *Expert Review of Respiratory Medicine*.

[5] Pérez-Rodriguez E., Castro D. J. (2000). The use of adenosine deaminase and adenosine deaminase isoenzymes in the diagnosis of tuberculous pleuritis. *Current Opinion in Pulmonary Medicine*.

[6] Zeng N., Wan C., Qin J. (2017). Diagnostic value of interleukins for tuberculous pleural effusion: a systematic review and meta-analysis. *BMC Pulmonary Medicine*.

[7] Kim C. H., Lee J., Lee J. (Oct 2015). Mycobacterial load affects adenosine deaminase 2 levels of tuberculous pleural effusion. *Journal of Infection*.

[8] Shibagaki T., Hasegawa Y., Saito H., Yamori S., Shimokata K. (1996). Adenosine deaminase isozymes in tuberculous pleural effusion. *The Journal of Laboratory and Clinical Medicine*.

[9] Gakis C. (Apr 1996). Adenosine deaminase (ADA) isoenzymes ADA1 and ADA2: diagnostic and biological role. *European Respiratory Journal*.

[10] Yanovich O. O., Titov L. P., Dyusmikeeva M. I., Shpakovskaya N. S. (2015). Evaluation of adenosine deaminase (ADA) and ADA1 and ADA2 isoenzyme activities in patients with pulmonary tuberculosis and tuberculous pleurisy. *International Journal of Mycobacteriology*.

[11] Andreasyan N. A., Hairapetian H. L., Sargisova Y. G., Mardanyan S. S., Badalyan L. T., Khanoyan A. S. (2002). Activity of adenosine deaminase and its isoforms in pleural fluid in tuberculous pleuritis. *Medical Science Monitor*.

[12] Moher D., Shamseer L., Clarke M. (2015). Preferred reporting items for systematic review and meta-analysis protocols (PRISMA-P) 2015 statement. *Systematic Reviews*.

[13] Whiting P. F., Rutjes A. W., Westwood M. E. (2011). QUADAS-2: a revised tool for the quality assessment of diagnostic accuracy studies. *Annals of Internal Medicine*.

[14] Jiang M., Li X., Quan X., Li X., Zhou B. (2018). Clinically correlated MicroRNAs in the diagnosis of non-small cell lung cancer: a systematic review and meta-analysis. *BioMed Research International*.

[15] Chu H., Guo H., Zhou Y. (2010). Bivariate random effects meta-analysis of diagnostic studies using generalized linear mixed models. *Medical Decision Making*.

[16] Reitsma J. B., Glas A. S., Rutjes A. W. S., Scholten R. J. P. M., Bossuyt P. M., Zwinderman A. H. (2005). Bivariate analysis of sensitivity and specificity produces informative summary measures in diagnostic reviews. *Journal of Clinical Epidemiology*.

[17] Deeks J. J., Macaskill P., Irwig L. (2005). The performance of tests of publication bias and other sample size effects in systematic reviews of diagnostic test accuracy was assessed. *Journal of Clinical Epidemiology*.

[18] Valdes L., San Jose E., Alvarez D., Valle J. M. (1996). Adenosine deaminase (ADA) isoenzyme analysis in pleural effusions: diagnostic role, and relevance to the origin of increased ADA in tuberculous pleurisy. *European Respiratory Journal*.

[19] Gorguner M., Cerci M., Gorguner I. (2000). Determination of adenosine deaminase activity and its isoenzymes for diagnosis of pleural effusions^∗^. *Respirology*.

[20] Tianrui-Xue G. C. (2005). Clinical investigation on diagnostic value of interferon-*γ*, interleukin-12 and adenosine deaminase isoenzyme for tuberculous pleurisy. *Chinese Medical Journal*.

[21] Mohammadtaheri Z., Mashayekhpour S., Mohammadi F., Mansoori D., Masjedi M. R. (2005). Diagnostic value of adnnosine deaminase isoznyme (ADA2) and total ADA in tuberculous pleural effusion. *Tanaffos*.

[22] Nalla N. K., Prasad C. E., Gopalakrishniah V., Somayajulu V. L., Chelluri L. K. (2009). Adenosine deaminase isoenzymes estimation - as a diagnostic tool for tuberculous pleural effusions. *Asian Pacific Journal of Tropical Medicine*.

[23] Zemlin A. E., Burgess L. J., Carstens M. E. (2009). The diagnostic utility of adenosine deaminase isoenzymes in tuberculous pleural effusions. *International Journal of Tuberculosis & Lung Disease*.

[24] Li F. Z., Gx H. (2009). Diagnostic value of adenosine deaminase isoenzyems in tuberculous pleurisy and carcinomatous pleurisy. *Clinics in Laboratory Medicine*.

[25] Shih W. S., Lin J. F., Sh L. (2011). Activities of adenine deaminase (ADA) and isoenzyme ADA2 in the pleural effusion from patients with TB pleurisy. *Journal of Biomedical & Laboratory Sciences*.

[26] Keng L. T., Shu C. C., Chen J. Y. P. (2013). Evaluating pleural ADA, ADA2, IFN-gamma and IGRA for diagnosing tuberculous pleurisy. *Journal of Infection*.

[27] Li M., Wang H., Wang X., Huang J., Wang J., Xi X. (2014). Diagnostic accuracy of tumor necrosis factor-alpha, interferon-gamma, interlukine-10 and adenosine deaminase 2 in differential diagnosis between tuberculous pleural effusion and malignant pleural effusion. *Journal of Cardiothoracic Surgery*.

[28] Yurt S., Küçükergin C., Yigitbas B. A., Seçkin S., Tigin H. C., Koşar A. F. (2014). Diagnostic utility of serum and pleural levels of adenosine deaminase 1–2, and interferon-*γ* in the diagnosis of pleural tuberculosis. *Multidisciplinary Respiratory Medicine*.

[29] Wang Xy Z. B. (2020). Differential diagnosis value of pleural effusion ADA, ADA2 and ACE levels in tuberculous and malignant pleural effusion. *Journal of Modern Laboratory Medicine*.

[30] Zhang Zj Z. Y. (2020). Diagnostic value of ADA and ADA isozyme in tuberculous pleurisy. *Hunan Normal University (Medical Science)*.

[31] Shen Y., Pang C., Shen K. (2016). Diagnostic value of thyroid transcription factor-1 for pleural or other serous metastases of pulmonary adenocarcinoma: a meta-analysis. *Scientific Reports*.

[32] Caraguel C. G. B., Vanderstichel R. (2013). The two-step Fagan’s nomogram: ad hoc interpretation of a diagnostic test result without calculation. *Evidence-Based Medicine*.

[33] Saghiri R., Ghashghai N., Movaseghi S. (2012). Serum adenosine deaminase activity in patients with systemic lupus erythematosus: a study based on ADA1 and ADA2 isoenzymes pattern. *Rheumatology International*.

[34] Gao Z.-W., Li R. C., Wang H. P. (2020). Diagnostic value of serum adenosine deaminase and its isoenzymes for autoimmune liver disease. *Hepatitis Monthly*.

[35] Aghaei M., Karami-Tehrani F., Salami S., Atri M. (2010). Diagnostic value of adenosine deaminase activity in benign and malignant breast tumors. *Archives of Medical Research*.

[36] Bielsa S., Palma R., Pardina M., Esquerda A., Light R. W., Porcel J. M. (2013). Comparison of polymorphonuclear- and lymphocyte-rich tuberculous pleural effusions. *International Journal of Tuberculosis & Lung Disease*.

[37] Andreasyan N. A., Hairapetyan H. L., Sargisova Y. G., Mardanyan S. S. (2005). ADA2 isoform of adenosine deaminase from pleural fluid. *FEBS Letters*.

[38] Hu Z. D., Liu X. F., Liu X. C., Ding C. M., Hu C. J. (2014). Diagnostic accuracy of osteopontin for malignant pleural mesothelioma: a systematic review and meta-analysis. *Clinica Chimica Acta*.

[39] Akobeng A. K. (2007). Understanding diagnostic tests 2: likelihood ratios, pre- and post-test probabilities and their use in clinical practice. *Acta Paediatrica*.

[40] Deeks J. J., Altman D. G. (2004). Diagnostic tests 4: likelihood ratios. *BMJ*.

[41] Ciledag A. K. A., Kaya A., Erol S. (2010). The comparison of pleural fluid TNF-α and IL-10 levels with ADA in tuberculous pleural effusion. *Current Medicinal Chemistry*.

[42] Inase N., Tominaga S., Yasui M., Tsukada Y., Oukouchi M., Miura H. (2005). Adenosine deaminase 2 in the diagnosis of tuberculous pleuritis. *Kekkaku: Tuberculosis*.

[43] Taganovich A. D., Alinezhad S. M. (2008). Diagnostic characteristics of adenosine deaminase test in Byelorussian patients with tuberculous pleurisy. *Problemy tuberkuleza i boleznei legkikh*.

